# PRECARITY AND WORK IN LATER LIFE: EXPERIENCES OF OLDER WORKERS IN UNCERTAIN TIMES

**DOI:** 10.1093/geroni/igad104.0886

**Published:** 2023-12-21

**Authors:** Cal Halvorsen, Christina Matz, Julie Miller

## Abstract

Employees later in their careers often earn the highest income of their lives, enabling them save for retirement while living in relative comfort. Yet many workers do not experience this late-career economic boost and instead face age bias and discrimination, difficulty finding and keeping work, and social and economic inequalities. Using an intersectionality lens, these difficulties often lead to disparities by race, ethnicity, gender, and immigration status, highlighting the varied socioeconomic experiences of older adults. The economic and social effects of the COVID-19 pandemic have often exacerbated these disparities. This symposium will highlight the varied experiences of late-career workers since the advent of the pandemic, with an emphasis on lower income workers and disparities by race and ethnicity. Dr. Choi-Allum will describe the results from a nationally representative survey of 2,000 workers ages 40 and older by AARP, highlighting how perceived age discrimination and the sense of a weak economy is driving uncertainty in finding and keeping paid work. Dr. Carr will identify racial and ethnic disparities in pandemic-related job disruption and its associated financial setbacks using Health and Retirement Study data. Dr. Halvorsen will describe the experiences of older, lower-income, and often immigrant job seekers within the federally funded Senior Community Service Employment Program in Massachusetts. And Dr. Matz will outline research with older adults who live in public housing on how social resources obtained partly through work may buffer financial strain’s impact on health. To conclude, Dr. Miller will place the four studies into the broader socioeconomic context. This is an Aging Workforce Interest Group Sponsored Symposium.

